# Does copy number variation of APOL1 gene affect the susceptibility to focal segmental glomerulosclerosis?

**DOI:** 10.1080/0886022X.2017.1323646

**Published:** 2017-05-11

**Authors:** Ting Peng, Guisen Li, Xiang Zhong, Li Wang

**Affiliations:** School of Medicine, University of Electronic Science and Technology of China, Renal Division and Institute of Nephrology, Sichuan Academy of Medical Sciences and Sichuan Provincial People's Hospital, Chengdu, China

**Keywords:** Focal segmental glomerulosclerosis, APOL1, copy number variation

## Abstract

**Background:** APOL1 risk variants (G1 and G2) are associated with increased susceptibility to focal segmental glomerulosclerosis (FSGS) in African population. However, the two risk mutations were not found in Chinese FSGS patients. In this study, we explored the association between the copy number variation (CNV) of APOL1 gene and FSGS.

**Methods:** APOL1 copy number variations were detected by quantitative real-time PCR with TaqMan probes and compared between 133 FSGS patients and 123 controls. The association between CNV of APOL1 gene and clinical parameters was also investigated.

**Results:** The distribution of APOL1 CNV did not show significant difference between FSGS patients and controls. The creatinine and proteinuria in the high copy number group (CN ≥ 3) were higher than the other two groups, but the difference was not significant (*p* > .05). The FSGS pathological types were different among the three groups.

**Conclusion:** There was no significant difference in the distribution of APOL1 gene copy variants between FSGS patients and normal controls, and there was no significant correlation between the APOL1 gene CNV and the FSGS patients’ clinical manifestations. APOL1 CNVs may be not associated with susceptibility to FSGS.

## Background

Focal segmental glomerulosclerosis (FSGS) is a glomerular disease characterized by diffuse fusion or effacement of podocyte foot processes [1] and is a common pathological type for resistant nephrotic syndrome (NS) in children and young patients [2]. Approximately, 40–70% of patients with FSGS would progress to end-stage renal disease (ESRD) [3,4]. Lots of studies demonstrated that genetic mutations could cause or increase susceptibility to FSGS by potentiating the effects of environmental factors [5–7].

The prevalence of FSGS in adult NS in African–Americans (AAs) is 2–3 times higher compared to Caucasian patients, and AAs with FSGS has a higher risk progression to ESRD [8]. The two risk variants (G1 and G2) of apolipoprotein 1 (APOL1) gene were predominantly present in African Americans and associated with 17-fold higher odds ratio for FSGS or increased risk of progression to ESRD [9–12]. However, none of the two risk mutations was found in Chinese population [13].

Copy number variation (CNV) is another kind of genetic variant and caused by the rearrangement of the genome. CNV generally refers to the length ranging from 1 kb to several megabytes for each copy, mainly for submicroscopic levels of deletion and duplication [14]. It presents in variable copy numbers compared to a reference genome [15] and plays a role in phenotypic diversity or evolution. Studies have revealed that the CNVs were associated with various human diseases, for example, low α-defensin gene copy number increases the risk for IgA nephropathy [16].

The copy number variation has been proved in APOL1 gene [17], but there was no report concerned about the relationship between CNV of APOL1 gene and FSGS. In this study, we explored whether the CNV of APOL1 gene could influence genetic susceptibility to FSGS or clinical manifestations of FSGS patients.

## Methods

### Subjects

A total of 133 adult (≥18 years old) sporadic patients with biopsy-proven primary FSGS were enrolled in this study. All patients with familial FSGS, HIV infection, intravenous drug abuse, glomerulomegaly due to other causes (such as obesity, sickle cell disease, cyanotic congenital heart disease, as well as diabetes) and FSGS secondary to reduced nephron numbers (such as unilateral renal agenesis and nephrectomy) were excluded. The average age of these patients including 87 males (65.4%) was 35.2 ± 17.8 years old. In total, 123 healthy individuals without known kidney diseases, hypertension, as well as diabetes were served as controls, including 43 males (35.0%). The average age of the controls was 52.0 ± 14.9 years old. All subjects’ genomic DNA was extracted from peripheral blood cells.

This study was approved by Institutional Review Boards of the Sichuan Academy of Medical Sciences and Sichuan Provincial People's Hospital. Written informed consents were obtained from all subjects prior to studies.

### Taqman gene copy number assay

The copy number assay was custom designed to amplify and detect a 142 bp region in the last exon of APOL1, and consisted of a forward primer (5′-TTACCAACTCACACGAGGCATT-3′), reverse primer (5′-CTCCACCTGTTCACCGCTTT-3′) and FAM labelled probe (5′-ACATCCGTGCCCTCA-3′) (Life Technologies). The 15 μL PCR mixture was consisted of 0.15 μL APOL1 probe, 0.3 μL forward and reverse primer, respectively, 0.75 μL RNAse P assay (reference assay, known to exist in two copies in human genome), 7.5 μL Taqman Master Mix, 3 μL molecular grade pure water and 3 μL sample DNA (50 ng/μL). Each sample was run in triplicate, and all the above reagents were obtained from Thermo Fisher Scientific (Thermo Fisher Scientific of China, Shanghai, China). The CNV assay was performed by using Applied Biosystems 7900HT quantitative real-time PCR system.

### Clinical data and renal pathology

Clinical data at renal biopsy were obtained from all patients, including age, gender, duration, serum albumin (Alb), serum creatinine (Scr), blood urea nitrogen (BUN), 24-h proteinuria. The serial sections of renal cortex biopsy with at least 10 glomeruli were included in this study. Sections were stained with haematoxylin and eosin (HE), periodic acid-schiff (PAS) together with silver methenamine and Masson’s trichrome. Immunofluorescence and electron microscopic descriptions from the reports were also reviewed. All the biopsies were independently revisited by one pathologist and one trained nephrologist. Glomerular sclerosis, tubular atrophy, interstitial fibrosis, as well as interstitial inflammation was semiquantitatively graded as negative (0), mild (1), moderate (2) or severe (3), which corresponded with 0%, 5–10%, 15–25%, 30–50%, respectively [18].

Primary outcome included [19]: (1) complete remission (CR): 24-h proteinuria <0.3 g, serum albumin ≥35 g/L, and serum creatinine did not exceed 25% of the baseline; (2) partial remission (PR): 24-h proteinuria decreased more than 50% of baseline value and less than 3.5 g per day, serum albumin increases or becomes normal and serum creatinine did not exceed 25% of baseline; (3) no remission (NR): Did not meet the above criteria were considered to be no remission.

### Statistical analysis

The Taqman copy number assay results were analysed using CopyCaller^®^ Software version 2.0 (Thermo Fisher Scientific Inc., Carlsbad, CA). The copy number (CN) call with 50% confidence was included in final analysis. Cases and controls were compared with respect to the result of their copy number (group 1: CN = 1; group 2: CN = 2; group 3: CN = 3 or 4). Comparisons among groups were performed with the chi-square test, Fisher’s exact test or ANOVA by using statistical software SPSS 19.0 (IBM Corp., Armonk, NY). The results were considered to be statistically significant if *p* < .05.

## Results

A total of 133 primary FSGS and 123 controls were included in this study, but there were two FSGS cases and one control with CNV typing failed. As showed in Table 1, 69.5% of FSGS patients and 75.4% of control groups had two copies, respectively. Although the proportion of two copies in controls is higher than in patients, the distribution of APOL1 CNV did not show significant difference between the two groups (17, 91, 22 or 1 in patients, and 12, 92, 17 or 1 in controls, for one-, two-, three- or four copy, respectively, *p* = .79).

**Table 1.** The copy number distribution in FSGS patients and controls.

	Copy number (CN)				
	1	2	3	4	*p*
FSGS (*n* = 131)	17	91	22	1	.79
Control (*n* = 122)	12	92	17	1	–

Totally, 121 primary FSGS with detailed clinical data were included finally for statistical analysis (Table 2). The reference copy number of APOL1 gene was 2 copies [17], and the main forms of copy number variation included the low copies (microdeletion) and multiple copies (microduplication) [20]. We divided the subjects into three groups according to the number of CN (Group 1: CN = 1, Group 2: CN = 2, Group 3: CN = 3 or 4).

**Table 2.** Demographic and clinical characteristics of the patients undergoing renal biopsy.

Parameters	Value (*n* = 121)
Age (years)	36.1 ± 16.6
Male, *n* (%)	82 (67.8%)
Duration (months)	28.17 (1, 24)
Hypertension, *n* (%)	23 (19.0%)
Oedema (0,1,2,3)	33/54/12/22
24-h proteinuria (g/24h)	6.65 (2.63, 9.03)
Serum creatinine (μmol/L)	115.88 (62.30, 133.00)
Serum albumin (g/L)	25.67 (16.00, 36.00)
Glomerular variants, *n* (%)	
No otherwise specified FSGS	74 (61.2%)
Cellular FSGS	9 (7.4%)
Tip lesion FSGS	30 (24.8%)
Collapsing FSGS	1 (0.8%)
Classic FSGS	7 (5.8%)

Oedema were graded as none (0), mild (1), moderate (2) or severe (3). All data were obtained at the time of renal biopsy.

There was no significant difference of the demographic and clinical parameters, such as age, gender, blood pressure, serum creatinine, as well as albumin (Table 3). Not otherwise specified (NOS), FSGS was the most common pathological type in the three groups (42.9%, 60.9%, 75%, respectively). There was significant difference (*p* = .014) for the distribution of the five pathological types in the three groups (Table 4). We also concerned about the glomerular sclerosis, tubular atrophy, interstitial inflammatory infiltration, as well as fibrosis, and we did not find significant differences for these pathological parameters in the three groups (Table 4).

**Table 3.** Comparison between the low copies group, normal copies group and multiple copies.

Parameters	Group 1 (*n* = 14)	Group 2 (*n* = 87)	Group 3 (*n* = 20)	*p*
Age (years)	38 ± 20	35 ± 17	38 ± 15	.68
Male, *n* (%)	10 (71.4%)	57 (65.5%)	15 (75.0%)	.68
Duration (months)	46.42	23.20	36.80	.35
Hypertension, *n* (%)	2 (14.3%)	17 (19.5%)	4 (20.0%)	.89
Systolic blood pressure (mmHg)	138 ± 18	137 ± 21	134 ± 15	.86
Diastolic blood pressure (mmHg)	89 ± 11	88 ± 15	82 ± 14	.36
24-h proteinuria (g/d)	6.26 (3.23, 9.95)	6.45 (2.38, 8.59)	7.93 (3.89,11.32)	.59
Serum creatinine (μmol/L)	93.57 (52.5, 133.5)	114.43 (61.98, 129.35)	136.58 (80.30, 138.50)	.34
Serum albumin (g/L)	23.57 (14.88, 32.85)	26.69 (17.15, 37.78)	22.69 (16.00, 26.5)	.27
Blood urea nitrogen(μmol/L)	7.96 (5.43, 11.11)	9.46 (4.65, 11.1)	10.06 (7.34, 12.05)	.69
Total cholesterol (mmol/L)	7.24 (4.70, 9.14)	8.54 (5.49, 10.99)	8.62 (5.60, 10.37)	.57
Uric acid (μmol/L)	320 (228, 410)	373 (278, 454)	334 (253, 432)	.25

All data were obtained at the time of renal biopsy.

**Table 4.** Comparison of pathological parameters among the groups with different copies of APOL1.

Parameters	Group 1 (*n* = 14)	Group 2 (*n* = 87)	Group 3 (*n* = 20)	*p*
Glomerular variants, *n* (%)				
No otherwise specified FSGS	6 (42.9%)	53 (60.9%)	15 (75.0%)	.014*
Cellular FSGS	0	9 (10.3%)	0	–
Tip FSGS	4 (28.6%)	23 (26.4%)	3 (15.0%)	–
Collapsing FSGS	1 (7.1%)	0	0	–
Classic FSGS	3 (21.4%)	2 (2.3%)	2 (10.0%)	–
Sclerosis (1/2/3)^a^	8/2/4	28/18/36	8/6/5	.53
Atrophy (1/2/3)^a^	3/1/3	19/13/9	2/3/1	.62
Fibrosis (1/2/3)^a^	3/3/0	19/12/11	1/3/4	.39
Inflammatory (1/2/3)^a^	5/5/0	22/12/14	2/2/5	.073

a 1 (0–10%); 2 (15–25%); 3 (30–40%).

* *p* < .05.

Forty-two patients were well followed up, 37 of which had two copies and the other five patients had copy number variation (one patient with CN = 1, and four patients with CN = 3) (Table 5). Considering that there is only one sample with one copy number, we just compared the normal copy number (CN = 2) with high copy number (CN = 3). There was no significant difference in remission rates between the two groups (*p* = .60).

**Table 5.** Comparison the influence on prognosis of FSGS between three copies and two copies (normal copies).

	CN =3 (*n* = 4)				CN =2 (*n* = 37)			
	CR	PR	TF	RR	CR	PR	TF	RR
CT	0	0	0	0	5	5	3	10/13
CTX	0	1	1	1/2	0	2	2	2/4
P	0	1	1	1/2	10	3	7	13/20
Total	0	2	2	2/4	15	10	12	25/37
Ratio				50%				67.6%

CR: complete remission; CT: conservative treatment; CTX: cyclophosphamide; P: prednisone; PR: partial remission; RR: remission rate; TF: treatment failure.

## Discussion

Many studies have confirmed that APOL1 gene mutations (G1 and G2) were associated with African sleeping sickness, atherosclerosis, as well as a variety of kidney diseases. Since Mathis [21] reported that FSGS could inherit in an autosomal dominant pattern, lots of studies have confirmed that the increased susceptibility of FSGS and ESRD in AAs may be mainly due to APOL1 genetic variants [22–25].

Variations in gene copy number were increasingly considered to be common and inheritable sources of individual differences in genome sequences. Recently, CNV was recognized as a common form of human genetic variation and contributor to a range of common diseases [26], such as systemic lupus erythematosus (SLE) [27] and cancer [28]. APOL1 located at chromosome 22, which was especially enriched in intrachromosomal duplications with approximately 100 kb present rearrangement [17]. In theory, a tandem duplication of APOL1 could extend its genetic effect to species [29], which could lead to change in gene dosage insufficient or excessive expression. Since G1 or G2 mutations were not detected in Chinese individuals [13], we could reasonably speculate whether CNV of APOL1 gene might affect individual susceptibility to Chinese FSGS.

We examined the CNV of the APOL1 gene in FSGS patients compared with healthy controls and determine the association between the CN of the APOL1 gene and clinical FSGS phenotypes. In this study, two copies of APOL1 gene accounting for about 70% in FSGS patients and healthy controls, and the distribution of APOL1 CNV was not significantly different between FSGS patients and healthy controls. Furthermore, there was no significant association between the CNV of APOL1 and clinical parameters of FSGS patients. These results suggested that CNV of APOL1 could not be related to the susceptibility and clinical manifestations of Chinese FSGS.

But there were several limitations that could influence the conclusion in this study. The first was the relative small sample size. Our sample size could differentiate the effect with odds ratio more than 2.0 (for disease in exposed subjects relative to unexposed subjects). A larger sample size would have a stronger statistical power. The second was that our study focused on adult FSGS. It could not deny the possible strong association between CNV and FSGS in children.

## Conclusions

In conclusion, CNVs of the APOL1 gene have been observed in both FSGS patients and healthy controls. Our study demonstrated that there were no significant association between APOL1 gene copy variants and the susceptibility to FSGS, or clinical manifestations of FSGS patients. APOL1 CNVs may be not associated with susceptibility to FSGS.
